# Reversibility of Age-related Oxidized Free NADH Redox States in Alzheimer’s Disease Neurons by Imposed External Cys/CySS Redox Shifts

**DOI:** 10.1038/s41598-019-47582-x

**Published:** 2019-08-02

**Authors:** Yue Dong, Sara Sameni, Michelle A. Digman, Gregory J. Brewer

**Affiliations:** 1 Department of Biomedical Engineering, University of California Irvine, Irvine, California United States of America; 2 Laboratory of Fluorescence Dynamics, Department of Biomedical Engineering, University of California Irvine, Irvine, California United States of America; 3 MIND Institute, Center for Neurobiology of Learning and Memory, University of California Irvine, Irvine, CA United States of America

**Keywords:** Senescence, Alzheimer's disease, Ageing, Cellular neuroscience

## Abstract

Redox systems including extracellular cysteine/cystine (Cys/CySS), intracellular glutathione/oxidized glutathione (GSH/GSSG) and nicotinamide adenine dinucleotide reduced/oxidized forms (NADH/NAD^+^) are critical for maintaining redox homeostasis. Aging as a major risk factor for Alzheimer’s disease (AD) is associated with oxidative shifts, decreases in anti-oxidant protection and dysfunction of mitochondria. Here, we examined the flexibility of mitochondrial-specific free NADH in live neurons from non-transgenic (NTg) or triple transgenic AD-like mice (3xTg-AD) of different ages under an imposed extracellular Cys/CySS oxidative or reductive condition. We used phasor fluorescence lifetime imaging microscopy (FLIM) to distinguish free and bound NADH in mitochondria, nuclei and cytoplasm. Under an external oxidative stress, a lower capacity for maintaining mitochondrial free NADH levels was found in old compared to young neurons and a further decline with genetic load. Remarkably, an imposed Cys/CySS reductive state rejuvenated the mitochondrial free NADH levels of old NTg neurons by 71% and old 3xTg-AD neurons by 89% to levels corresponding to the young neurons. Using FLIM as a non-invasive approach, we were able to measure the reversibility of aging subcellular free NADH levels in live neurons. Our results suggest a potential reductive treatment to reverse the loss of free NADH in old and Alzheimer’s neurons.

## Introduction

Neurons rely on redox couples to buffer oxidative and reductive stress using millimolar concentrations of extracellular Cysteine (Cys)/Cystine (CySS) and intracellular glutathione (GSH)/ glutathione disulfide (GSSG). Kinetically, intracellular glutathione is maintained by reduced nicotinamide adenine dinucleotide (NADH)/oxidized (NAD^+^) and reduced nicotinamide adenine dinucleotide phosphate (NADPH)/oxidized (NADP^+^). Each redox couple is compartmentalized and somewhat independent with distinct redox pools in the mitochondria, cytoplasm and nuclei^1,2^. Maintaining an independent mitochondrial GSH pool size is critical to preserve a reducing environment for mitochondrial proteins against oxidative DNA damage^3^. The crosstalk of reduced components between nuclear and other compartments has been demonstrated importantly in cell cycle progression^4^ and redox signaling^5,6^. Additionally, the NADH/NAD^+^ redox states impact the regulation of gene expression^7^ by affecting the activity of transcription factors^8^ and cellular signaling^9^. Thus, systematic studies of changes in the redox environment with aging could inform age-related bioenergetic aspects of neurodegeneration.

Age is the most important risk factor for Alzheimer’s disease (AD). Aging promotes the oxidation of redox couples. In human plasma, aging is associated with the oxidation of the GSH/GSSG as well as the Cys/CySS redox state after middle-age^10^. In rat brain, NAD(P)H levels decrease after middle age^11^. In brain neurons, NADH is kinetically upstream of GSH^12^ and therefore, manipulating NADH levels influences GSH levels. Free NADH is generated from a cytoplasmic dehydrogenase in glycolysis (glyceraldehyde 3-phosphate dehydrogenase, GAPDH) and mitochondrial dehydrogenases (pyruvate dehydrogenase, PDHC, and other the TCA cycle dehydrogenases). The produced free NADH is used to power oxidative phosphorylation for ATP generation. NADH exists in two states, either in a protein-bound form or a free form. Our previous study used steady-state fluorescence imaging without being able to distinguish between bound and free NADH^13^. Studies of imposed Cys/CySS redox states demonstrated the feasibility to manipulate internal NAD(P)H levels^12^ in mouse neurons and internal glutathione (GSH) levels of human retinal pigment epithelial cells^14^. We recently reported an age-related decrease in intracellular free NADH of brain neurons^15^. Since vulnerability to external stress and energetic shortage constitute two of the principal manifestations of aging, here we non-invasively measured free NADH in mitochondria, cytoplasm and nuclei of aged neurons at various redox states. In order to examine the capacity to maintain free NADH levels, we imposed an external Cys/CySS oxidative stress. More importantly, we tested whether free NADH can be restored to young levels by imposing an extracellular reductive state. We applied a non-invasive and sensitive imaging technique to measure the intrinsic NADH fluorescence. Using fluorescence lifetime imaging microscopy (FLIM), we distinguish the bound and free NADH based on the long and short intrinsic fluorescent lifetimes of NADH^16,17^ and also resolve subcellular compartments for free NADH re-distribution in response to an imposed external oxidative and reductive states in live neurons.

## Results

### Imposed extracellular Cys/CySS redox states modulate intracellular NADH levels

**Figure 1:**
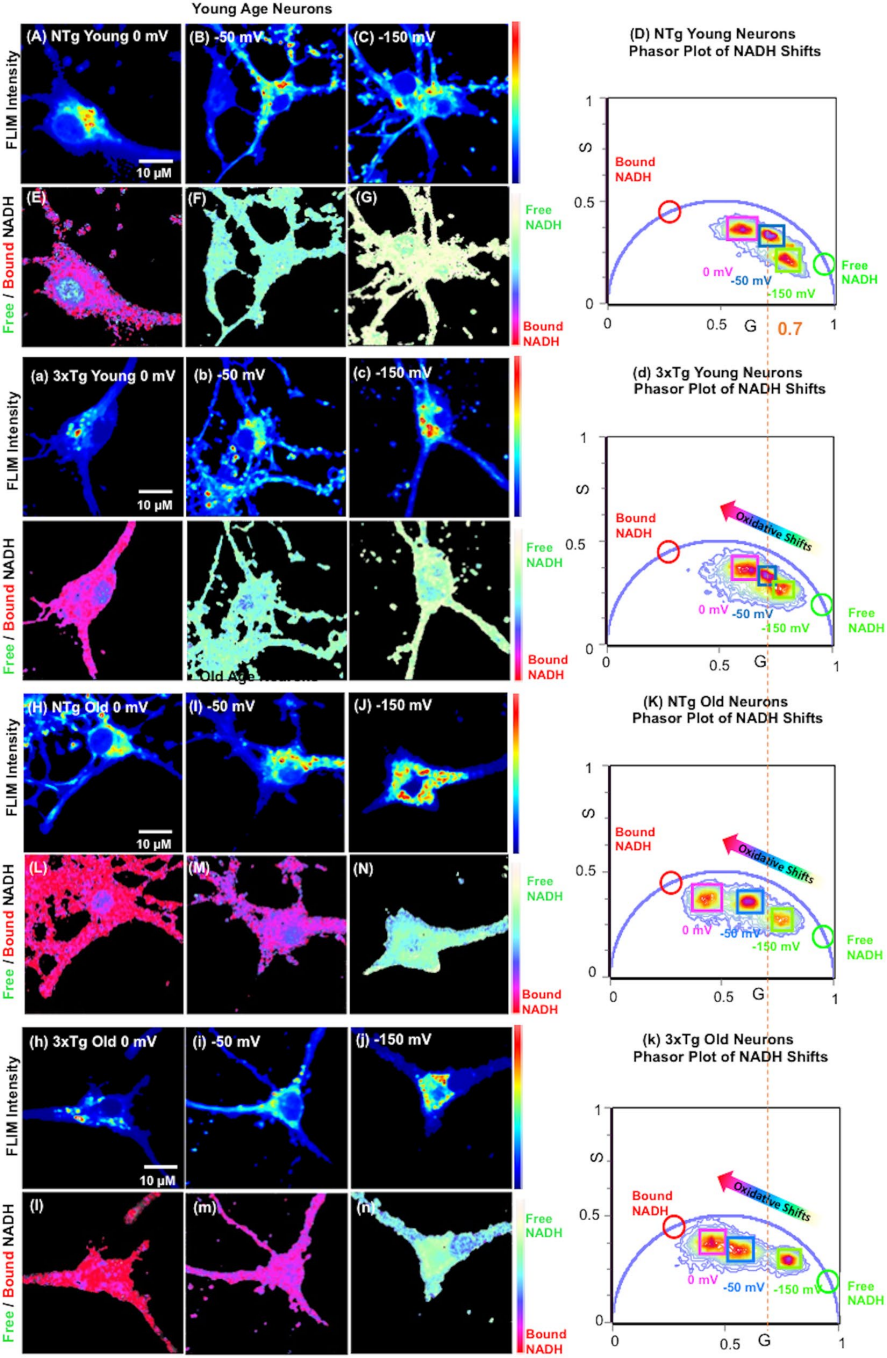
Imposed external Cys/CySS oxidative and reductive states shift the internal NADH redox state in neurons from NTg and 3xTg-AD female mice. FLIM NADH Intensities were collected from NTg (**A**–**C**) and 3xTg-AD (a–c) in young age neurons under 0 mV (oxidative), −50 mV (normal medium as control, no treatment) and −150 mV Cys/CySS (reductive) redox states. (**D**) Phasor plot of the same NTg and (d) 3xTg-AD young age neurons illustrate the shifts of the intracellular free/bound NADH ratios toward more free NADH at reductive −150mV and to more bound NADH with imposed external oxidative state of 0 mV. Each cluster represents NADH signals from pixels of the corresponding single neuron on the left. The colored arrow indicates the direction of shifts from free to bound NADH with imposed reductive to oxidative states. Corresponding free /bound NADH FLIM color maps of the NTg (**E**–**G**) and 3xTg-AD (e–g) of young age neurons demonstrate intracellular NADH shifts to higher free/bound NADH proportions with an external reductive shift showing more cyan-green. In the FLIM fraction colormap, pink-purple regions indicate more bound NADH and green-cyan refers to more free NADH fraction. An imposed oxidative stress to 0 mV shifted the cellular distribution of NADH free/bound ratio toward more red-purple, indicating a shift to a lower free/bound NADH fraction. Similarly, FLIM NADH intensities were collected in old age neurons from NTg (**H**–**J**) and 3xTg-AD mouse neurons (h–j) following the indicated redox treatments. Phasor plot of the same NTg (N) and (n) 3xTg-AD old neurons shows the manipulation of intracellular free/bound NADH ratios of neurons in response to the imposed external Cys/CySS oxidative and reductive states. The dashed vertical orange line indicates the G value at the center of NTg young cluster at the −50 mV (normal) Cys/CySS condition for reference. Corresponding free and bound NADH distributions from old neurons of NTg (**L**–**N**) and 3xTg-AD (l–n) mice with imposed redox states.

Age-associated oxidative stress lowers both total (bound + free) NADH levels^11,18^ and free NADH in neurons^15^ in varying proportions. To study free NADH in live neurons as a function of age and transgenic AD genotype, we isolated and cultured neurons in a uniform medium from mice at young, middle and old ages, removed from a complicated aging context *in vivo* including age-related inflammation, hormones and vasculature. To study how the external redox shifts impact intracellular free NADH levels and distributions, we applied a 120 fs pulsed laser excitation for fluorescence lifetime imaging microscopy (FLIM) to distinguish free NADH from bound forms (Fig. 1) at a normal balance of extracellular Cys/CySS (−50 mV), an imposed oxidative state (0 mV, excess CySS) and an imposed reductive treatment (−150 mV, excess Cys). The FLIM intensity images of neurons attest to the ability of withstand measurements with a Cys/CySS oxidative and reductive state in young NTg neurons **(**Fig. 1A–C**)** and 3xTg-AD neurons **(**Fig. 1A–C**)**, compared to old NTg **(**Fig. 1H–J**)** and 3xTg-AD neurons **(**Fig. 1H–J**)**. Mitochondria were localized and selected by imaging TMRE fluorescence^15^. Since each pixel contains information of the lifetimes of free and bound NADH molecules, we transformed the signal from each pixel onto a phasor plot to visualize the proportions of free and bound in young age neurons of NTg (Fig. 1D) and 3xTg-AD (Fig. 1D) as well as old age neurons of NTg (Fig. 1K) and 3xTg-AD (Fig. 1K). In the phasor plot, the coordinates G and S represent the real and the imaginary part of the fast Fourier transformation (FFT) respectively^19^. As a combination of free and bound lifetimes, the cellular signal clusters along the line between the bound NADH lifetime of 3.4 ns (red circle) and pure free NADH lifetime of 0.4 ns (green circle). In response to the imposed oxidative Cys/CySS redox states, the NADH cluster from a whole neuron shifted toward more bound NADH, relative to the cluster of the untreated condition in both young (Fig. 1D,d) and old neurons of both genotypes (Fig. 1K,k). Under the imposed reductive states, the neuronal NADH lifetime cluster shifted to more free NADH compared to the untreated condition. In the free and bound NADH distribution map **(**Fig. 1E–G**)**, the cyan-green color refers to more free NADH while red-purple indicates more bound NADH. Neurons treated with an oxidative condition showed a shift in NADH fraction from free to more bound NADH (Fig. 1E,e,L,l). In contrast, an imposed external reductive state caused a shift in cellular distribution toward more free NADH (Fig. 1G,g,N,n).

### Mitochondrial free/bound NADH proportions respond to imposed oxidative and reductive states

**Figure 2:**
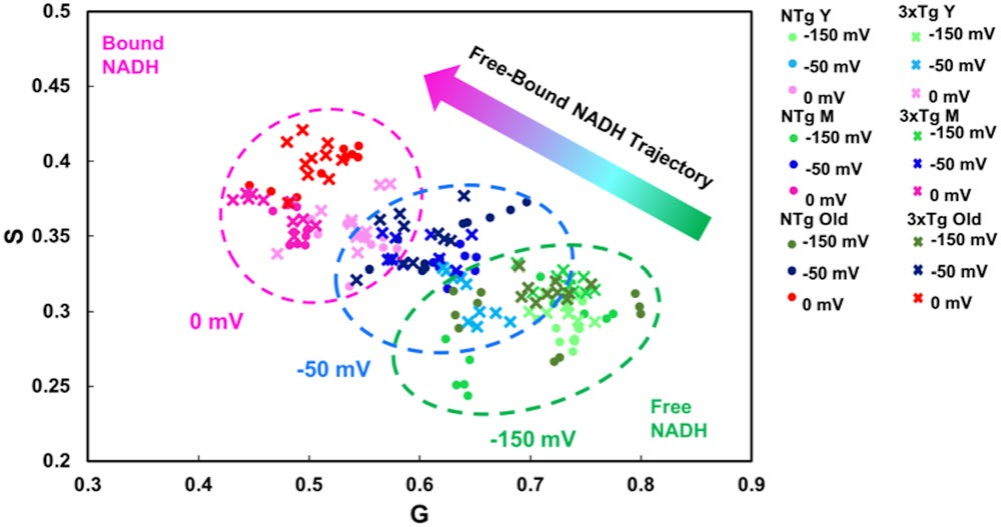
Mitochondrial NADH shifts toward more bound NADH with oxidative states and toward more free form in response to the imposed external reductive treatment (female neurons). Each dot or cross point represents one neuron, the average of mitochondrial regions (ROIs) from a single NTg or 3xTg-AD neuron (n = 10 neurons/age/genotype/redox state = 180 points). The dashed ellipses are manual groupings by imposed redox state. Note that mitochondrial free/bound NADH re-distributes in response to the manipulated external Cys/CySS redox states from reductive (−150 mV) to oxidative (0 mV) in both genotypes and three ages are shown by the colored arrow (ANOVA for G, F(2, 179) = 547, p < 0.001; for S, F(2, 179) = 356, p < 0.001).

Free NADH levels in mitochondria are critical for complex I to maintain the proton gradient across the mitochondrial membrane to power ATP production in the process of oxidative phosphorylation (OXPHOS). We used 2-photon excitation microscopy to resolve mitochondrial free and bound proportions in the NADH pool. We determined whether the mitochondrial free NADH levels can be manipulated with imposed extracellular Cys/CySS oxidative and reductive states. Figure 2 shows (G, S) data plotted from 10 cells of each age and genotype as a function of external oxidative and reductive shifts. In general, for both genotypes and 3 ages, the extracellular oxidative stress (pink circle) caused a shift toward more bound NADH relative to untreated mitochondria. The imposed external reductive state (green circle) caused dramatic shifts toward higher free NADH in the (G, S) plot of all ages and both genotypes. These results indicate the ability to shift mitochondrial free and bound NADH fractions of all ages and both genotypes by imposed external redox shifts.

### imposed external Cys/CySS redox states modulate NADH at mitochondria, cytoplasm and nuclei by age, AD-genotype and gender

Given that the age- and AD-associated declines in free NADH levels^15^, we investigated (1) age- and AD-related differences of compartment-specific free NADH proportions in response to the external oxidative stress and further determine if old age diminishes the capacity to maintain free NADH against external imposed oxidative stress; (2) More importantly, whether the lower free NADH levels in old neurons could be restored by imposed external Cys/CySS reductive state in either genotype. Note that measures of the free NADH fraction are relative to the total NADH pool size (free + bound). Therefore, as the total NADH pool size diminishes with age and AD, computed changes in absolute free NADH concentrations could be larger than the changes in free NADH fraction of the total. From these measures of free NADH fraction, we further measured the free NADH concentrations in mitochondria across the age span to adjust for different total NADH pool sizes. We describe each of these effects first for mitochondria at each redox state, then cytoplasm and nucleus in terms of age, genotype and sex effects.

#### Mitochondrial free NADH fractions

**Figure 3:**
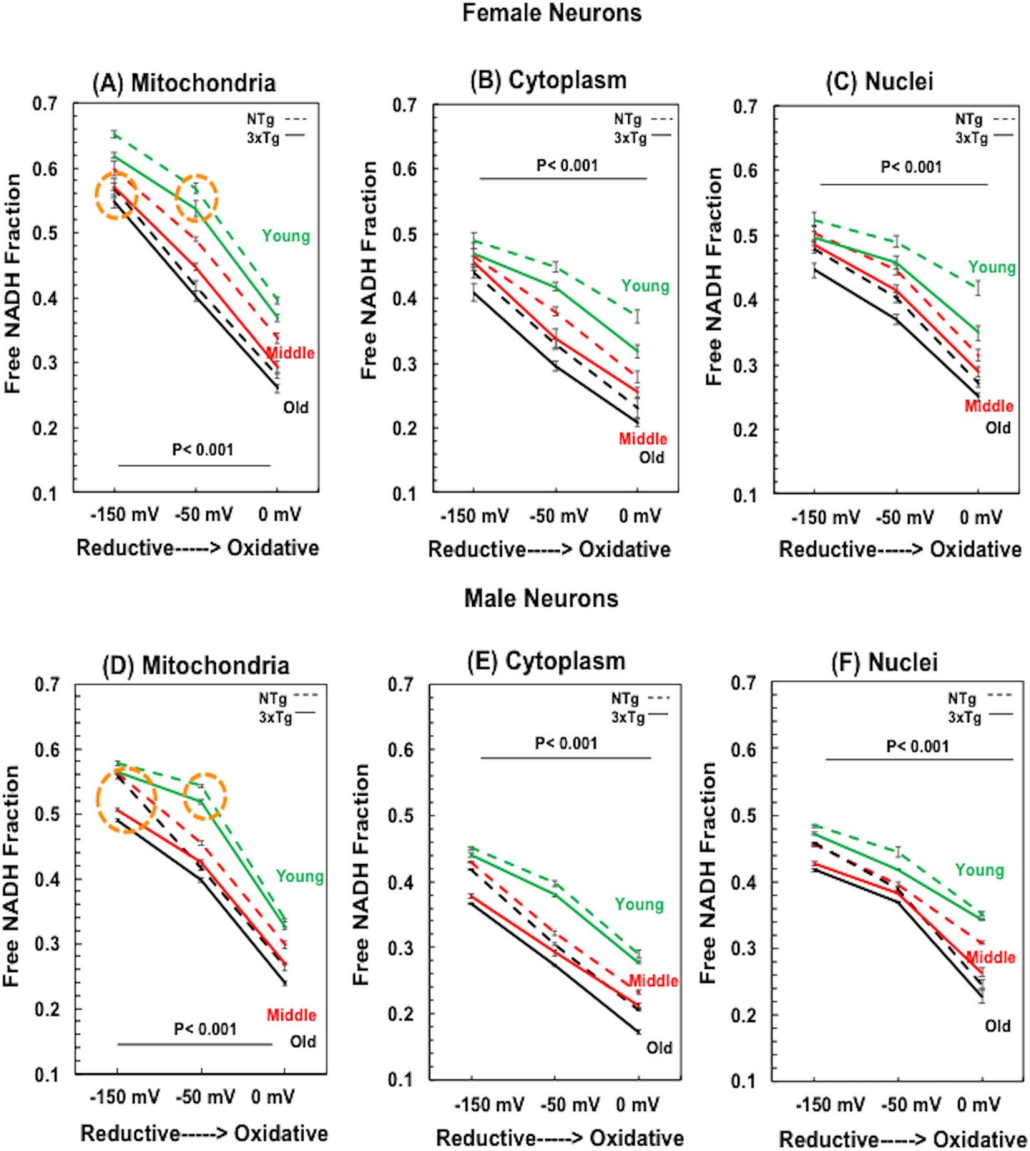
Compartment-specific changes in free NADH fractions with imposed extracellular oxidative (0 mV) and reductive (−150 mV) states across ages of NTg and 3xTg-AD mice for both female (**A**–**C**) and male (**D**–**F**) neurons. (**A**,**D**) Mitochondrial free NADH fractions in response to the imposed external Cys/CySS reductive states at −150 mV and imposed oxidized state at 0 mV in all ages (p < 0.001) of both NTg and 3xTg-AD mice. With external reductive treatment, mitochondrial free NADH fractions can be rescued to the levels of young age at untreated condition (orange circles). Untreated condition of −50 mV was a control for comparison. At the untreated condition, age effect drove declines in mitochondrial free NADH from young to old age of each genotype (p < 0.001). Moreover, free NADH fractions with genetic load appear to be lower than that of age- and redox- matched NTg neurons (p < 0.001). The imposed oxidative stress depleted free NADH fractions in all ages (p < 0.001). Notably, mitochondrial free NADH fractions in young age always presented highest than the levels of middle and old age in response to the external redox shifts. (**B**,**E**) Cytoplasmic free NADH fractions and (**C**,**F**) Nuclear free NADH fractions of female neurons illustrated depletion with oxidative stress (p < 0.001) and elevation in reductive states (p < 0.001) across ages in NTg and 3xTg-AD mice. Similarly, in male neurons. For gender difference, male neurons exhibited lower free NADH fractions than the age-, genotype- and redox-matched female neurons in each compartment, (Mito. F(1, 59) = 13, p < 0.001; Cytoplasm F(1, 59) = 25, p < 0.001; Nuclei F(1, 59) = 13, p < 0.001); This sex-driven redox orders remain unchanged with external oxidized stress, (Mito. F(1, 59) = 33, p < 0.001; Cytoplasm F(1, 59) = 24, p < 0.001; Nuclei F(1, 59) = 5, p = 0.03) and with the imposed reductive state (Mito. F(1, 59) = 22, p < 0.001; Cytoplasm F(1, 59) = 3.7, p = 0.06; Nuclei F(1, 59) = 22, p < 0.001); Genotype effect drove a lower free NADH fraction in 3xTg-AD neurons than the NTg neurons in each age, redox state and compartment (n = 20 female neurons or n = 10 male neurons/compartment/redox state/age/genotype).

At 0 mV Oxidative State: The imposed external Cys/CySS oxidative stress (0 mV) caused declines in mitochondrial free NADH fractions in all ages (Two Way ANOVA for ages, female F(2, 119) = 165, p < 0.001 and male F(2, 59) = 112, p < 0.001) and genotypes (Two Way ANOVA for genotypes, female F(1, 119) = 35, p < 0.001 and male F(1, 59) = 26, p < 0.001) (Fig. 3A,D). Interestingly, under the external oxidative stress, the declines of mitochondrial free NADH fractions from −50 to 0 mV of all ages and genotypes ranged narrowly from 30–35% in female (Fig. 3A) and 37–40% in male neurons (Fig. 3D). Specifically, at the 0 mV oxidative condition the old-age free NADH fraction were 21–30% lower than young neurons in mitochondria of both genders and genotypes. However, since the old neurons started at a 23–26% lower free NADH fraction, at 0 mV the old neurons were shifted closer toward a viable limit of free NADH.

At −150 mV Reductive State: To determine if the lower free NADH fractions in old age neurons can be rescued by a reductive treatment, we imposed an extracellular Cys/CySS reductive state of −150 mV in the culture media. Figure 3A,D show that this reductive stress elevated mitochondrial free NADH fraction in all ages, genotypes and genders compared to the untreated conditions (Two Way ANOVA for age, female F(2, 119) = 37, p < 0.001 and male F(2, 59) = 60, p < 0.001) and genotypes (Two Way ANOVA for genotypes, female F(1, 119) = 13, p < 0.001 and male F(1, 59) = 164, p < 0.001). Under the imposed reductive state, mitochondrial free NADH of young neurons increased 6–15% and old neurons rose by 23–26% for both genotypes and genders. The external reductive state enhanced the mitochondrial free NADH fraction in old neurons to values approaching the those of young age under the untreated condition (orange circles) of both genotypes and genders. This indicates the lower mitochondrial free NADH in old age can be restored with an external reductive shift. Additionally, successful rejuvenation of the mitochondrial free NADH fractions in old age neurons suggests a reversible latent capacity to regenerate free NADH in old age neurons. With external reductive state, the lesser 6–15% rise of free NADH fractions in young than the 23–36% in old age neurons suggests an approach toward a maximal capacity for free NADH regeneration.

Manipulability: Over the range of imposed oxidative to reductive conditions, the mitochondrial free NADH fractions of young neurons could be manipulated by 64–74% and old age neurons were affected 105–111% (treatment*age interaction for female F(4, 359) = 9, p < 0.001; for male F(4, 179) = 36, p < 0.001) for both genotypes and genders. The higher manipulability in old age neurons suggests a higher vulnerability to resist external redox stress and a lower capacity to maintain mitochondrial free NADH levels.

#### Cytoplasmic and Nuclear Compartment-specific Changes in Free NADH Fractions

To explore compartment-specificresponses in free NADH fractions with extracellular reductive and oxidative stress, we further measured free NADH fractions in cytoplasm **(**Fig. 3B,E**)** and nuclei **(**Fig. 3C,F) for all ages, both genotypes and genders. In the untreated condition, cytoplasmic free NADH fractions of old neurons were 23–29% lower than young neurons for both genotypes and genders (treatment*age for female F(4, 359) = 8, p < 0.001; male F(4, 179) = 19, p < 0.001). Nuclear differences in free NADH fractions at the untreated redox state were 11–19% lower in old than young neurons (treatment*age in nuclei for female F(4, 359) = 11, p < 0.001; male F(4, 179) = 20, p < 0.001). Under the imposed external oxidative stress to 0 mV, cytoplasmic free NADH fractions decreased 17–27% in young and 30–37% in old age neurons of both genotypes and genders. Similar to that of mitochondria, an external imposed reductive shift to −150 mV nearly restored both cytoplasmic and nuclear free NADH fractions in old age neurons to the levels of young neurons under the untreated condition. Of note, over the entire redox range, the manipulability of nuclear free NADH fractions by external redox shifts was 25–43% in young and 77–87% in old neurons of both genotypes and genders. These percentages are lower than the changes in the cytoplasm of old-age (91–103%) or the mitochondria (105–111%), suggesting a stronger capacity in nuclei to maintain free NADH levels relative to the total NADH pool size and minimize impacts on changes in free NADH with external redox stress. Remarkably, mitochondria were the most reduced with highest free NADH fractions, followed by more oxidized nuclei and most oxidized cytoplasm. This order was affected little by the imposed reductive and oxidized conditions, genotype age or gender.

#### More Oxidized State in Male Neurons than Female Neurons

Females have a higher incidence of AD than males as well as other neurodegenerative diseases^20,21^. In response to redox shifts, we found the free NADH fractions to be consistently higher in female than male neurons across the age-span in both genotypes and three compartments (gender effects F(1, 1619) = 375, p < 0.001; treatment*gender*compartment interaction F(4, 1619) = 3, p = 0.02). Under the same external oxidative and reductive stress, the free NADH fraction in female neurons was 5–15%, 6–22% and 3–16% higher than that of male in mitochondria, cytoplasm and nuclei respectively. The largest gender-difference of free NADH fractions was found in cytoplasm, suggesting a more glycolytic metabolism in female neurons under various external redox states.

### Free NADH concentrations and total NADH pool size in mitochondria with imposed redox states

**Figure 4:**
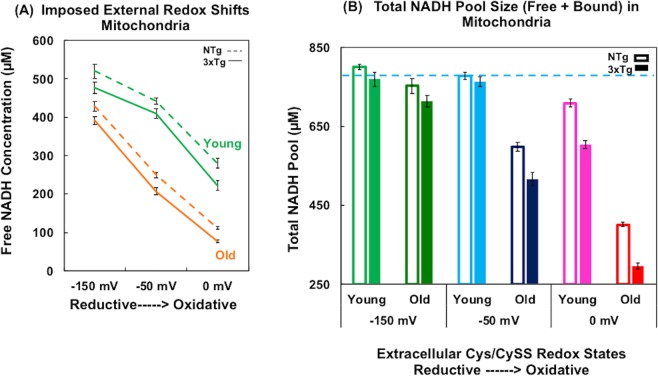
Absolute free NADH concentrations and total NADH pool size shift in mitochondria in response to an imposed external Cys/CySS reductive (−150 mV) or oxidative (0 mV) state for young and old age neurons from NTg and 3xTg-AD female mouse brains. (**A**) Under the imposed external oxidative stress, free NADH in mitochondria of both young and old neurons, both genotypes was depleted compared to that of untreated condition at −50 mV (at 0 mV, NTg young F(1, 31) = 112, p < 0.001; NTg old F(1, 31) = 338, p < 0.001; 3xTg young F(1, 31) = 128, p < 0.001; 3xTg-AD old F(1, 31) = 198, p < 0.001) and was elevated with external reductive state to −150 mV (at −150 mV NTg young F(1, 31) = 16, p < 0.001; NTg old F(1, 31) = 154, p < 0.001; 3xTg young F(1, 31) = 13, p = 0.0013; 3xTg-AD old F(1, 31) = 183, p < 0.001) (n = 16 neurons/treatment/age/genotype). (**B**) Mitochondrial total NADH pool size in each redox state of female of both genotypes. As a reference, the blue dashed line indicates the total NADH pool in young NTg neurons at −50 mV redox state.

As the NADH pool size changes with age, genotype, redox environments and energetic states, free NADH fraction reflects a change of free NADH level relative to the current NADH pool size (free + bound). To assess absolute free NADH concentrations in mitochondria of live neurons, we adjusted the FLIM phasor measures for the lower quantum yield of the free and higher quantum yield of the bound form of NADH^22^. Under oxidative stress of 0 mV, in NTg neurons (Fig. 4A), mitochondrial free NADH concentrations of young age neurons declined by 37% to 280 µM (F(1, 31) = 112, p < 0.001) and old age neurons decreased 55% to 110 µM (F(1, 31) = 338, p < 0.001) compared to the untreated condition (−50 mV). In 3xTg-AD neurons, the free NADH concentration in young neurons declined 46% (F(1, 31) = 128, p < 0.001) and that in old neurons dropped 63% (F(1, 31) = 198, p < 0.001) compared to the untreated condition (−50 mV). AD genetic load drove a further 10% depletion in mitochondrial free NADH concentrations compared to the age-matched NTg neurons. This indicates that with external oxidative stress, old neurons had lower capacity to maintain mitochondrial free NADH concentrations than the young neurons of both genotypes. Furthermore, neurons with the genetic load were even lower than NTg neurons in their capacity to conserve mitochondrial free NADH in response to the oxidative stress.

With imposed external reductive state to −150 mV, the mitochondrial free NADH concentration of old neurons was elevated by 71% to 430 µM in NTg (F(1, 31) = 598, p < 0.001) and by 89% to 390 µM in 3xTg-AD neurons (F(1, 31) = 183, p < 0.001). Under the reductive environment, the free NADH levels in old neurons approach the levels in young neurons of 442 µM in NTg and 410 µM in 3xTg-AD at the untreated condition (−50 mV). The successful rejuvenation of mitochondrial free NADH concentrations in old neurons under the external reductive treatment indicates a latent capacity to restore free NADH back to the young levels of both genotypes. However, reductive treatment on young neurons only drove a 18% increase of mitochondria free NADH concentrations in NTg (F(1, 31) = 16, p = 0.0004) and 16% elevation in 3xTg-AD neurons (F(1, 31) = 13, p = 0.0013) respectively. This suggests an approach to the maximal capacity for free NADH regeneration (or minimal consumption) in the young age with this reductive condition at −150 mV. Though the increment was smaller in young neurons with the reductive shift, the mitochondrial free NADH concentrations were still 22% higher than the old neurons with reductive treatment in both genotypes.

From the range of external oxidative to reductive stress, the overall flexibility for alteration of mitochondrial free NADH concentration in old neurons was 30% higher that of young neurons in both genotypes (n = 16 neurons/treatment/age/genotype) (redox treatment*age interaction F (2, 191) = 25, p < 0.001). This suggests that old neurons were more susceptible to external redox stress than young neurons. By comparison of the pattern of young and old differences in Figs 4A to 3A for the same neurons, the percent changes driven by age in mitochondrial free NADH concentrations in Fig. 4A were 2-fold larger than that in free NADH fractions in Fig. 3A. Figure 4B indicates the reason as a smaller NADH pool (free + bound) size in old neurons compared to young neurons at each redox state (ANOVA for treatment*age F(2, 191) = 53, p < 0.001; treatment*genotype F(2, 191) = 8, p = 0.001). The commonly reported free (or bound) NADH fraction can be compared to that of free (or bound) NADH concentrations only if the NADH pool is constant (free + bound NADH). Figure 4B shows large changes in the NADH pool with age and genotype under each redox state. Thus, the changes in free NADH concentration can be larger than the fractional changes because of these changes of NADH pool. The smaller size of the NADH pool in old-age was exacerbated by an external oxidative stress but can be nearly restored to the untreated young levels by a reductive shift to −150 mV.

## Discussion

Free mitochondrial NADH provides the major source of energy in neurons. The redox environment is critical for regulating NADH and NAD levels^23^. How aging influences the free NADH regenerating capacity of mitochondria is less clear. Here for the first time we have reported the reversibility of free NADH levels even in old age and AD with an external oxidative or reductive stress. By using a non-invasive method of FLIM, we were able to quantify free and bound NADH fractions, concentrations and pool sizes in live neurons in response to the modified external redox states and distinguish separate responses in neuronal mitochondria, cytoplasm and nuclei. With 6 hours of an external oxidative shift in Cys/CySS redox state, the intracellular free NADH levels were lowered in mitochondria, cytoplasm and nuclei of all ages, approaching levels needed for survival^13^. Subcellular free NADH in old neurons moved lower than the that in young age neurons of both genotypes, suggesting a lower capacity to preserve free NADH in old age. Additionally, the 3xTg-AD genotype resulted in further lowering of levels of free NADH compared to that of age-matched NTg neurons. Remarkably, a reductive shift could rejuvenate free NADH in old neurons to the levels of untreated young neurons. We propose that the extracellular reductive Cys/CySS states not only enhance the internal free NADH concentrations, but provide substrate energy for enzymatic functions to reverse the energetic deficit in aging and AD. Though the redox systems and signaling of each compartment remain somewhat independent, the imposed Cys/CySS reductive states dramatically rescued the loss of capacity in regenerating free NADH of old age neurons in mitochondria, cytoplasm and nuclei of both genders and genotypes. Under all redox states, we observed that mitochondria had higher NADH levels than nuclei or cytoplasm, indicating a compartment-specific distribution of free NADH levels. This reductive privilege of mitochondria enables maximum generation of ATP from feeding free NADH to the electron transport chain. Our findings suggest the potential for reductive treatments in aging and AD to reverse the course of oxidative shifts.

Our fluorescence lifetime imaging methods yielded NADH concentrations with minimal disturbance to the neurons. In Table 1, we compare the NADH concentrations that we obtained by FLIM for mitochondria to those obtained from whole cells or cell or tissue extracts. Since mitochondrial concentrations of NADH are higher than cytoplasmic, whole cell or tissue measures are generally lower than our mitochondrial measures. Overall, the method of measurement and calibration results in large variations in concentrations, but the FLIM method is likely to be the least perturbing and most representative of the native state.

**Table 1:** Cellular NADH concentrations.

		Free NADH Concentration (µM)			Total NADH Pool (free + bound) (µM)			Method	Reference
**A**. **Neuronal Mitochondria**									
Mice	Imposed redox state (mV)	**0**	**−50**	**−150**	**0**	**−50**	**−150**	FLIM	This work
NTg female hippocampus	young	280	441	520	708	777	799		
	old	112	250	428	401	598	753		
3xTg female hippocampus	young	223	410	476	603	762	769		
	old	77	207	391	295	515	713		
Mouse brain mitochondria	young					1500		HPLC	^58^
**NAD**									
Mouse neuron culture	young		470					Enzymatic cycling	^59^
**B**. **Whole Neurons**									
Mice					**0**	**−50**	**−150**	Steady state fluorescence	^13^
NTg male hippocampus	young				49	79	78		
	old				38	77	81		
3xTg male hippocampus	young				43	61	61		
	old				29	33	48		
Rat brain CA1 homogenates	young				800–1300			Enzymatic cycling	^60^
Rat	middle					250		HPLC	^11^
	old					110			
Male mouse hippocampus	3 mo					57			^61^
**C**. **Other cells**									
Normal breast cell line(Hs578Bst)						99		FLIM	^16^
Breast cancer cell line (Hs578T)						168			
Rat Liver	3 mo					1500	µmol/kg protein	thiazolyl blue micro-cycling	^62^
	12 mo					1650			
	24 mo					3750			
Heart	3 mo					4050			
	12 mo					4500			
	24 mo					6300			
Kidney	3 mo					150			
	12 mo					165			
	24 mo					750			
Lung	3 mo					143			
	12 mo					146			
	24 mo					735			
Rat liver mitochondrial						3500		HPLC	^63^

Protein cysteine residues serve as redox molecular switches to signal regulatory and adaptive responses^24^. A prototypic instance is given by the Kelch-like ECH-associated protein 1 (Keap1)- nuclear factor erythroid 2-related factor 2 (Nrf2)-antioxidant response elements (ARE) (Keap1-Nrf2-ARE) pathway. An oxidized redox state causes an oxidation of cysteines in Keap1 to promote dissociation from Nrf2^25^. This allows Nrf2 to translocate to the nucleus to promote transcription of antioxidant response genes^26^. As the critical feature of reversible thiol redox switches, we investigated whether an imposed external Cys/CySS reductive state could reverse the age- and AD-related loss of intracellular free NADH levels in neurons. In support, dietary supplementation of glutathione precursors cysteine and glycine in elderly subjects can dramatically drive a 95% increase in glutathione concentration and 79% elevation in fractional synthesis rate in plasma^27^. Protective effects on cysteine-rich proteins were seen in dietary supplementation with SelenoCysteine, which is also available from milk proteins and egg to improve antioxidant status^28^.

Though intracellular free NADH levels decline with age and AD-genotype, our data suggests that the free NADH levels in old age NTg and AD-model neurons can be restored back to the young age levels by the external manipulation of Cys/CySS redox circumstances. This restoration to youthful levels of free NADH could provide the reductive energy for higher rates of oxidative phosphorylation. Higher ATP flux could explain the improved survival of these old neurons with reductive shifts through a mechanism of higher pAKT^13^. More generally, redox biology interfaces the individual exposome (e.g. diet and exercise; here redox shift) and functional metabolome and genome^29^. Our imposed redox shifts are important because extracellular redox states play critical roles in numerous biological processes including proliferation^30^, differentiation^31^ and apoptosis^32^. Systemically, the thiol/disulfide couples of cysteine/cystine (Cys/CySS) and glutathione/glutathione disulfide (GSH/GSSG) are in thermodynamic disequilibrium in plasma^33^. Inside cells, the GSH/GSSG couple buffers the redox state, but NADH is kinetically upstream of GSH^12^. Cys residues and most thiols in proteins can be kinetically manipulated for redox control^33,34^.

Our results demonstrate that the free NADH concentration and NADH pool are under direct and rapid redox control by external Cys/CySS oxidative and reductive shifts. The reductive shift in the circadian rhythm of plasma Cys/CySS with eating^35^ indicates the opportunity for *in vivo* manipulation. These results suggest a potential use of redox-based therapy as an effective tool to delay or even reverse the course of aging and AD by reversing the depletion of free NADH levels to improve ATP generating capacity. Here we only evaluated one level of reductive shift and less reductive shift may be necessary to avoid reductive stress^36^. *In vivo*, this will require titration to biomarker targets. In previous work, neuronal (free + bound) NAD(P)H levels were compared after titrated inhibition of NAD(P)H regeneration or inhibition of glutathione re-synthesis, which consumes NADPH^12^. The results in old neurons indicated that regeneration of NADH was more important than consumption of NAD(P)H for regeneration of glutathione.

Our studies were motivated by reported changes in the Cys/CySS redox state in human plasma which was progressively oxidized with age 0.2 mV per year from 18 to 93 years^10^. In neurons, the excitatory amino acid transporter EAAT3 facilitates entry of the zwitterionic amino acid L-cysteine^37^. The cystine/glutamate antiporter SLC7A11/xCT is also responsible for uptake of extracellular cystine and release of intracellular glutamate^38,39^. The activity of EAAT3 maintains the intracellular pool of free cysteine for glutathione synthesis^40^. Addition of extracellular L-cysteine elevates intracellular GSH levels^36^. CySS is immediately reduced to Cys in the more reducing cytoplasm^41^. However, the mechanism for increasing intracellular free NADH levels by the imposed reductive Cys/CySS states remains unclear. If NADH also serves as an anti-oxidant, then higher levels of GSH could relieve some ROS-dependent consumption of NADH. Additionally, NADH is in equilibrium with NADPH via transhydrogenase^9^ and malate enzyme^42^. NADPH is used for reductive energy by glutathione reductase to regenerate GSH from GSSG. Therefore, a reductive shift that elevates GSH^13^, would lessen consumption of NADPH and thus NADH. A reductive shift may indirectly improve the free NADH levels by reducing the oxidized protein thiols and reversing the activities of thiol-containing redox couples and enzymes. Cysteine residues at active sites of proteins, such as thioredoxin (Trx), glutaredoxin (Grx) and peroxiredoxin (Prx), dominate oxidative detoxification^43^. These characteristics facilitate a reversible redox switch in response to the extracellular reductive and oxidative circumstances. These responses have been especially well documented for NMDA receptors as protein cysteines dimerize in an oxidized redox state to limit Ca^++^ flux in aging hippocampal neurons^44^. As a fast response to the environmental stimuli, reversible changes of protein thiols provide rapid post-translational switches in control of the functions of mitochondrial proteins^45^.

Clinical attempts to modulate cysteine were reported recently. Placebo-controlled clinical trials with *N*-acetylcysteine (NAC) as the supplement in psychosis patients showed significant improvements on neurocognition and increase of GSH levels in brain and blood cells^46,47^ as well as working memory performance^48^. NAC functions as a powerful antioxidant for detoxification because of its role as a precursor of L-cysteine for glutathione biosynthesis^49^. In fibroblasts of AD patients, the combinatorial intervention of lipoic acid (1 mM) and NAC (100 µM) pronouncedly decreased mitochondrial-related oxidative stress^50^. Due to its safety as a nutritional supplement, NAC was also examined for AD treatment in clinical trials. In some clinical trials, L-cysteine is used as an auxiliary treatment for AD patients. A phase II randomized clinical trial of a nutraceutical formulation (including NAC, α-tocopherol, acetyl-L-carnitine, folate, B12, S-adenosyl methioinine) on 106 AD patients showed a stable or improved cognitive performance and mood/behavior^51^. Administration of a supplement cocktail of antioxidants including L-cysteine, vitamin E, vitamin C, β-carotene, selenium, vitamins B1, 2, 3, 6, 9 and 12 may augment pharmacological approaches in AD treatment^52^. In this study, we presented the successful reversibility of oxidized free NADH in mitochondria of old age neurons to the young levels from NTg and 3xTg-AD mouse brains by imposing an externally reductive Cys/CySS shift to −150 mV. This provides additional motivation for therapy for AD by administration of reductive cysteine redox states *in vivo*.

Free NADH functions critically to power energetic supply and maintain intracellular redox homeostasis. In an advance over previous *in vitro* NADH studies of brain aging and Alzheimer’s, this work used FLIM, to discriminate free and bound NADH in segregated mitochondrial, cytoplasmic and nuclear compartments of live primary neurons in response to the external imposed Cys/CySS oxidative and reductive manipulation. Our method is label-free and non-invasive probing the intrinsic fluorescence of NADH in live neurons by two-photon excitation. Since the total NADH pool size varied by age, genotype and external redox shifts and free NADH fraction is relative to the total NADH pool size, a further quantification of free NADH concentrations and total NADH pool sizes in mitochondria facilitated comparison of their capacity to maintain or regenerate free NADH. With external reductive treatment, the free NADH fractions in old neurons can be reversed and rejuvenated to the levels of young age, indicating a potential reductive intervention to reverse or slow down the progression of aging and AD. The measured free NADH concentrations suggests a lower capacity in maintaining or regeneration of free NADH in old neurons than the young neurons and a further diminish with genetic load in the age-matched 3xTg-AD neurons. Additionally, in line with the compartmental redox order in HeLa cells^53^, we found in neurons, regardless of external redox states, mitochondria displayed the most reduced with highest free NADH fractions, followed by nuclei and most oxidized in cytoplasm with lowest free NADH fractions. The successful reversibility of free NADH concentrations in old age neurons back to the levels of healthy young age by an external reductive state suggests a potential reductive intervention to counter AD and extend lifespan.

## Methods

### Mouse model

As described before^15^, we used LaFerla’s triple transgenic mouse model of AD (3xTg-AD) with human transgenes *βAPP* (SWE), *PS1* (M146V), and *Tau* (P301L) to mimic the neuropathological features of AD^54^. Nontransgenic (NTg) C57BL/6/ were used as controls. All mice underwent genotyping before using in experiments. All experiments involving mice were approved by the Institutional Animal Care and Use Committee (AUP-17-65) and performed according to guidelines and regulations.

### Primary neuron culture

As describe previously^55^, adult hippocampal neurons were isolated from NTg and 3xTg-AD age-matched mouse brains at young (3–4 months old), middle ages (9–10 months old) and old ages (18–23 months old). Hippocampi were sliced at 0.5 mm, digested with papain (Worthington) and triturated in Hibernate A (BrainBits LLC, Springfield, IL) with 2% B27 supplement (Fisher Scientific) with 0.5 mM Glutamax (Fisher Scientific). Neurons were separated from debris and microglia on an Optiprep (Sigma-Aldrich) density gradient. The neuron-enriched fraction was collected. Neurons were plated at 50,000 cells/cm^2^ on 15 mm glass coverslips and cultured in NbActiv1 (BrainBits) with 5 ng/mL mouse FGF2 and 5 ng/mL PDGFbb (Fisher Scientific) for trophic support. Prior to plating, glass coverslips (Assistant; Carolina Biological) were coated overnight with 100 µg/mL poly-D-lysine. Neurons were cultured for 9–12 days at 37 °C in 5% CO_2_, 9% O_2_ at saturated humidity. Viability in our neuronal cultures was similar to previous studies of neurons from all ages and both genotypes^15,18^. The neuronal densities of all ages and genotypes in culture were similar, without fragmented axons or dendrites, indicating the capacity to withstand the imposed redox shifts. The mitochondria of neurons were prelabeled with 10 nM TMRE (tetramethylrhodamine ethyl ester) for 20 minutes under 5% CO_2_ at 37 °C^15^. In the same fields as used for FLIM, mitochondria were imaged by 561 nm laser excitation and 597–737 nm filtered emission. Nuclear subcellular regions were selected by their central circular appearance. Cytoplasmic sub-regions were chosen in regions lacking mitochondria in TMRE images.

### Variation of Cys/CySS redox state

We controlled the external redox state in the culture medium with an excess of either cysteine or cystine. The B27 medium also contains redox-active superoxide dismutase, catalase, glutathione and vitamin E^56^ as redox buffers. Fresh stock solutions of 10 mM cysteine and 5 mM cystine were made in culture medium NbActiv1 (BrainBits) each time. The Cys/CySS redox state for NbActiv1 is −50 mV. As described before^13^, oxidized and reduced Cys/CySS redox states were modulated by varying cysteine and cystine concentrations based on the Nernst equation: Eh (mV) = −250 + 30 log ($CySS/[Cys]$^2^). The modulated reductive and oxidative potentials of Cys/CySS were made by adjusting the proportions of Cys and CySS stock solution in the NbActiv1 medium. The final medium concentration of Cys at the −150 mV redox state was 180 µM (without CySS) and the final concentration of CySS at 0 mV was 100 µM (without Cys). Neurons were incubated under different Cys/CySS redox states for 6 hours at 37 °C in 5% CO_2_, 9% O_2_. In our previous work^13^, we measured the external redox potential by HPLC to confirm the Nernst calculation at the start. Jiang group measured the time-dependent changes in concentrations of Cys and CySS and E_h_ levels in retinal pigment epithelial cells^14^. During 5 hours of treatment with extracellular 200 µM Cys, the Cys/CySS redox potential dropped from −158 mV to around −80 mV, measured by HPLC.

### Free NADH calibration

According to Ma *et al*.^22^, the β-Nicotinamide adenine dinucleotide (NADH, Sigma-Aldrich) was prepared fresh each time as a stock solution of 428 µM in a 0.2 mM Tris-HCl, pH 7.5 and stored at 4 °C. The absolute concentration of the free NADH stock solution for calibration was determined prior to use in a NanoDrop 2000 UV-Vis Spectrophotometer (Thermo Fisher Scientific Inc) at 340 nm. The actual free NADH concentration was calculated based on the Beer-Lambert law

$${\boldsymbol{A}}={\boldsymbol{\varepsilon }}{\boldsymbol{bc}}$$

where ***A*** is the readout absorbance; **ε** is the extinction coefficient of NADH (6.22 at 340 nm); **b** is the length of light path (1 mm) and **c** refers to concentration of NADH in the solution.

### FLIM Imaging

As described previously^15^, fluorescence lifetime images were acquired at 37 °C in 5% CO_2_ on a Zeiss LSM 710 microscope (Carl Zeiss, Jena, Germany) using a 63x oil immersion objective, 1.2 N.A. (Carl Zeiss, Oberkochen, Germany). The 2-Photon excitation laser source was a Titanium:Sapphire MaiTai laser (Spectra-Physics, Mountain View, CA) with 120 fs pulses and 80 MHz repetition rate. Pixel dwell time was 25.21 µs and image size was 256 × 256 pixels. The cultured neurons were excited at 740 nm employing an emission band pass filter at 460/80 nm. The signal (autofluorescence) was collected with a photomultiplier tube (H7422P-40, Hamamatsu, Japan).

### FLIM phasor and sub-regional data analysis

Analyses were performed using a phasor method^57^ and SimFCS software (LFD,UCI). From Stringari^17,19^, every pixel of the FLIM image was transformed to one pixel in the phasor plot by a Fast Fourier Transform (FFT) of the intensity decay I(t). The coordinates g and s in the phasor plot are the real and imaginary part of the FFT by using the following transformations:

$${g}_{i,j}(\omega )=\frac{{\int }_{0}^{t}{I}_{i,j}(t)cos(\omega t)dt}{{\int }_{0}^{t}{I}_{i,j}(t)dt}$$

$${s}_{i,j}(\omega )=\frac{{\int }_{0}^{t}{I}_{i,j}(t)sin(\omega t)dt}{{\int }_{0}^{t}{I}_{i,j}(t)dt}$$

where the indices *i* and *j* identify a pixel of the image and ω represents the frequency (ω = 2πƒ), with ƒ is the laser repetition rate (80 MHz) and t is the period of the laser, 12.5 ns. Based on the linearity of the phasor coordinates, the g and s position of each pixel represents the fraction of free to bound NADH in the image^19^.

To study the free NADH levels of mitochondria, regions of interest (ROI) were selected based on TMRE staining of mitochondria^15^. Cytoplasmic regions replete of mitochondria were selected. Circular nuclear regions were readily evident from their low NADH signal. Five ROIs were measured in each compartment of each neuron. Measurements of compartments from 20 female neurons and 10 male neurons from each age and genotype mouse group were averaged.

### Measurements of total NADH Pool and Free NADH concentrations in mitochondria

Before calibration with free NADH for absolute NADH quantification, we calibrated the instrument response function with a known standard, 100 µM Coumarin 6 with lifetime at 2.5 ns. In the phasor plot, the pure free NADH lifetime is 0.4 ns^22^. Whereas, the NADH bound to LDH (lactate dehydrogenase) has a longer lifetime of 3.4 ns. The lifetime distribution of the NADH signal from the cells represents the fractional combination of free and bound NADH along the line between the pure free NADH and bound NADH. To measure the total NADH pool in the neurons, we first acquired a FLIM image of a known concentration of free NADH calibrated using the absorbance spectrophotometer. We then corrected for the difference between a lower quantum yield of the free and higher quantum yield of the bound form of NADH as described by Ma *et al*.^22^. To determine the mitochondrial free NADH concentration, we multiplied the measured total NADH pool by the corresponding free NADH fraction in mitochondria.

Lifetime per se is independent of fluorophore concentration. However, the fraction of two lifetimes depends on the fractional contribution of the two fluorophores. The method described by Ma *et al*.^22^, uses this concept where one of the components is a constant intensity (such as a “lamp” intensity) of phasor position at 0,0. By adding this component we can measure the concentration of the other fluorophores. The method requires the measurement of a solution of free NADH at a known concentration. The phasor representation of the fluorescence decay accounts for the differences in quantum yield of the free and bound form of NADH, pixel by pixel of an image. The concentration of NADH in every pixel in a cell is obtained after adding to each pixel in the phasor plot a given amount of unmodulated light which causes a shift of the phasor towards the origin by an amount that depends on the intensity at the pixel and the fluorescence lifetime at the pixel. In our case we are measuring the fractional contribution of the free and bound NADH cofactors. For each of these two components, using the different amounts of unmodulated light as a calibration for the phasor at 0,0 lifetime allows us to calculate the absolute concentration of the bound and free NADH.

### Statistical analysis

One-way or Two-way ANOVA analysis was used to assess the difference of means and variances in Excel. Multifactorial ANOVA analysis was conducted in IBM SPSS Statistics software. The level of significance was set at p < 0.05 to reject the null hypothesis. Means and SEs are presented.
